# Advanced glycation end products as a source of artifacts in immunoenzymatic methods

**DOI:** 10.1007/s10719-017-9805-4

**Published:** 2018-01-05

**Authors:** Aleksandra Kuzan, A. Chwiłkowska, K. Maksymowicz, A. Bronowicka-Szydełko, K. Stach, C. Pezowicz, A. Gamian

**Affiliations:** 1 Department of Medical Biochemistry, Faculty of Medicine, Wrocław Medical University, ul. T. Chałubińskiego 10, 50-368 Wrocław, Poland; 2 Department of Forensic Medicine, Medical Faculty, Wrocław Medical University, ul. J. Mikulicza-Radeckiego 4, Wrocław, Poland; 3 Department of Biomedical Engineering, Mechatronics and Theory of Mechanisms, Faculty of Mechanical Engineering, Wrocław University of Technology, ul. Łukasiewicza 7/9, 50-371 Wroclaw, Poland; 4 L. Hirszfeld Institute of Immunology and Experimental Therapy, Polish Academy of Sciences, Weigla 12, 53-114 Wrocław, Poland

**Keywords:** Glycation, Extracellular matrix, Collagen, Elastin, Immunoenzymatic methods

## Abstract

The most abundant proteins in the arteries are those of extracellular matrix, ie. collagen and elastin. Due to their long half-lifes these proteins have an increased chance to undergo glycation. The aim of this study was to determine relationship between the content of the main extracellular matrix proteins and the advanced glycation end products (AGEs) in arteries. In this study 103 fragments of aorta were analyzed by ELISA and immunobloting for the content of collagens type I, III and IV and elastin and the content of advanced glycation end-products (AGE). A negative correlation between the content of collagens type III and IV and AGE (*r* = −0,258, *p* = 0,0122, and a weak negative correlation between collagen type III and age of the sample donor (*r* = 0,218, *p* = 0,0262) were demonstrated. This result comes as a surprise and it contradicts an intuitive assumption that with more glycation substrate, *i.e*. matrix proteins, more AGE products are expected. We have concluded that the results of the ELISA tests must have been influenced by the glycation. As a consequence, either modified protein molecules were not being recognized by the antibodies, or the glycation, and formation of crosslinks have blocked access of the antibodies to the antigen. It will conceal the effect of the linear dependence between the result (absorbance/densitometry) from the quantity of protein to which the antibody is directed.

## Introduction

Glycation is a non-enzymatic process, where reducing sugars (glucose, fructose, glucose-6-phosphate and other) react with amino groups of proteins. It occurs spontaneously inside and outside of living organisms. Since only a small portion of sugars is present in a chain form with accessible free aldehyde group, this process under physiological conditions occurs very slowly, over period of weeks or months [1]. Therefore, any negative outcome of the glycation concerns proteins with a long half-life, such as collagen and other extracellular matrix proteins [2]. This process appears to intensify in people suffering from diabetes. Formation of the crosslinks between the adjacent protein molecules, leads to their decreased solubility and reduced susceptibility to enzymatic digestion. Furthermore, modification of the amino acid side chain in the filament proteins alters distribution of the electrostatic charges and structural changes that may skew interactions with other proteins [3]. Collagen and elastin are key structural proteins defining biomechanical properties of the arteries. They ensure strength (collagen) and elasticity (elastin), both with extremely long half-lifes, *i.e*. approx. 70 years [4]. It is known that they constitute over 50% of the dry weight of the artery [5] and that the quantity of this tissue may be modulated by different processes, for example atherogenesis. Still, it is unclear whether the amount of collagen and elastin grows, or falls with age. Hardening of the arteries in elderly suggests that the amount of collagen increases and the amount of elastin decreases with age and progression of the atherosclerosis, but experimental data do not allow for unambiguous confirmation of such hypothesis [6–10]. We have previously described the relationship between collagen type II and the degree of atherosclerosis [11] and among other types of collagen and degree of disease (unpublished data). This is work where our focus is on the products of advanced glycation associated with extracellular matrix proteins.

ELISA and immunoblotting were main biochemical techniques used for the determination of ECM proteins. These two methods are highly specific and sensitive, (detection of less than 1 ng of protein). These methods are based on the following principle: primary antibody reacts with a specific epitope present on the targeted protein, followed by the reaction with secondary antibody conjugated with an enzyme (in this case horseraddish peroxidase - HRP). Colorimetric detection of reaction product catalizated by HRP allows for quantitative analysis of the antigen in tissue extract. Immunocytochemical and immunohistochemical tests are based on the same principle. These methods, however, are not free from methodological problems. This work aims at highlighting the source of artefacts that can distort determination of certain antigens, in particular proteins those with long half-life, *i.e*. collagen and elastin.

## Materials and methods

### Material

Material consisted of fragments of 103 aortic abdominal or thoracic segment, collected post mortem at the Department of Forensic Medicine at the Wroclaw Medical University in 2010–2013. The samples came from individuals who died of a sudden death, aged 55 +/− 15 years, 74 men and 29 women. The storage time of the preparations from their collection to testing did not exceed 12 h.

## Methods

### ELISA

Arterial samples weighting 40 mg each were homogenized with FastPrep-24® (MP Biomedicals) in 700 μl of extraction buffer (Tris 10 mM, EDTA 5 mM, NaCl 0.2 M, pH 7.5). The homogenate was then diluted 200-fold with PBS (137 mMNaCl, 2.7 mMKCl, 8.1 mM Na_2_HPO_4_, 1.5 mM KH_2_PO_4_). MaxiSorp 96 plates (Nunc) were coated with antigen – collagen type I, III, IV or elastin present in homogenates. Each sample was tested in three replicates. In parallel, the procedure was carried out for the standard solution of collagen type I (Millipore, CC050) at concentrations of 6, 3, 2, 1, 0.75, 0.5, 0.25, 0.1, 0.05 μg/ml, for the standard solution of type III collagen (Millipore, CC054) at concentrations of 1, 0.5, 0.25, 0.125, 0.064, 0.032 μg/ml, for the standard solution of collagen type IV (Millipore, CC076) at concentrations of 1, 0.5, 0.25, 0.125, 0.064, 0.032 μg/ml, for the standard solution of elastin (Sigma, E6902) at concentrations of 8, 4, 2, 1, 0.5, 0.25, 0.125 μg/ml. After coating for 5 h at room temperature, plates were blocked using 10% skimmed milk in TPBS at 4 °C overnight. Then antibodies were applied – mouse IgG1 anti-collagen type I (Novus Cat# NB 600-450, RRID:AB_522923) diluted 1:2000, mouse monoclonal anti-collagen type III (Sigma-Aldrich Cat# C7805, RRID:AB_476874) diluted 1:4000, mouse monoclonal anti-collagen type IV (Sigma-Aldrich Cat# C1926, RRID:AB_476828) at a dilution of 1:2000, rabbit polyclonal anti-elastin (Santa Cruz Biotechnology Cat# sc-25736, RRID:AB_640101) at a dilution of 1:4000. Incubation of antibodies was carried out at room temperature for 2 h. The secondary antibody either rabbit anti-mouse IgG (H + L) conjugated with peroxidase (Jackson ImmunoResearch Labs Cat# 309-035-082, RRID:AB_2339653) at a dilution of 1:5000, or goat anti-rabbit IgG conjugated to peroxidase (Jackson ImmunoResearch Labs Cat# 111-035-045, RRID:AB_2337938) diluted 1:3000 were applied. The incubation proceeded at room temperature for 1.5 h. Then peroxidase was allowed to react with substrate – *o*-phenylenediamine hydrochloride (OPD, Sigma) and absorbance at 450 nm was read on Enspire plate-reader (Perkin Elmer).

### Glycation of collagen and elastin

To confirm the research hypothesis, additional experience was made by performing the ELISA test on the standards of analyzed collagen and elastin in form of glycated and control proteins. 10–6 μg of type I collagen / type III collagen / type IV collagen and elastin were left under favorable glycation conditions: in the presence of 400 mM glucose-6-phosphate in PBS at 37 °C for 11 days. Verification of glycation was obtained by measuring the fluorescence of samples at excitation wavelength 350 nm and emmision 440 nm and performing a fluorescence spectrum at excitation wavelength 350 nm and emission range 370–500 nm. Next, a ELISA test analogous to the above described was done, by performing standard curve with the glycated protein. Parallel test was performed with untreated protein by glycation.

### Dot blot analysis

Tissues were homogenized on ice in MilliQ water using a FastPrep homogenizer-24^®^ (MP Biomedicals) keeping the ratio of water to tissue 1 μg/10 μl. Homogenate was diluted 200 times with PBS. In the next step 800 μl of homogenate was applied on a PVDF membrane (Immobilon®-P Transfer Membrane, Millipore, pore size 0.45 μm, IPVH00010) using a slot apparatus (Bio-Dot® SF Microfiltration Apparatus, Bio-Rad). On each membrane 8 slots were filled with increasing concentrations of the synthetic AGE standard to give a calibration curve in the range 0–1.6 μg. The AGE product corresponding to the antigen to which antibody were directed, was obtained by high temperature microwave synthesis and isolated by liquid chromatography (column with HW-55S gel in 0.01 M ammonium acetate buffer) [12]. The membrane was immersed in 5% skim milk in TBST and incubated at room temperature for 2 h and at 4 °C for 18 h. Membranes were then washed three times with TBST and treated with a solution of primary noncommercial antibodies: anti-AGEmel10 at 1:100 dilution in TBS. Antibodies and standard are also described in Leszek *et al*. [13]. After 2.5 h incubation at room temperature, membranes were washed again and treated with 10,000-fold diluted solution of anti-mouse IgG conjugated with peroxidase (Jackson ImmunoResearch Labs Cat# 111-035-045, RRID:AB_2337938). Sample that was not allowed to react with primary antibodies was used as a reference. Membranes were washed with TBST and incubated with peroxidase substrate AEC (3-amino-9-ethylcarbazole, Sigma, A5754). After 5 min, the membrane was rinsed with distilled water. Intensity of the obtained bands was analyzed by densitometry using the Gel Doc apparatus (Gel Doc ™ XR+ Gel Documentation System, Bio-Rad). While using standard curves the amount of AGE in homogenates was calculated and then converted to 1 mg of tissue.

### Statistical analysis

Statistical analysis was performed using Spearman’s correlation method and multivariate regression. The R statistical package Windows version 3.1.2 was used for statistical analysis. Significance was accepted at *p* < 0.05.

## Results

ELISA analysis revealed that the artery wall contains on average of 19.38 (+/− 11.74) mg/g of tissue collagen, 15.85 (+/− 10.12) mg/g of tissue elastin and 0.522 (+/− 0.373) mg/g tissue of the AGE antigen. Results shown in Table 1 were analyzed for the correlation between AGE and tested proteins, taking into account the particular types of collagen.

**Table 1:**
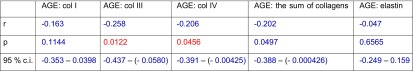
Spearman correlation calculated for the content of the advanced glycation end-products (AGE) and extracellular matrix proteins

Statistically significant correlations are shown in red

*col*, collagen; *r*, Spearman correlation coefficient; *p*, significance level, *95% c.i*, 95% confidence interval

There has been no significant correlation between the contents of the advanced glycation end-products and the content of collagen type I, the sum of collagens and elastin. However there was a weak negative correlation between the amount of AGE and the content of collagen type III and IV in arteries samples. This is surprising, since it was to be expected that the more the glycation substrate, which is collagen and elastin, the more advanced glycation end products. The negative correlation indicated an inverse relationship.

The correlation between the age of the person from whom the sample came from and the protein content of the ECM and the AGEs was calculated. The results are shown in Table 2. No statistically significant correlation was found between the content of collagens type I and type IV, elastin, sum of collagens and the age of the sample donors. However, there is a statistically significant correlation between the content of type III collagen and the age, indicating that with age there is a tendency to the reduction of the type III collagen content in the aorta.

**Table 2:**
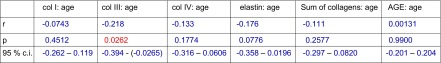
Relationship between the content of various extracellular matrix proteins (collagen types I, III, IV, elastin), advanced glycation end products and the age of the person from whom tissue sample has originated, as analyzed by Spearman correlation method

Statistically significant correlations are shown in red

*col*, collagen; *r*, Spearman correlation coefficient; *p*, significance level; *95% c.i*, 95% confidence interval

**Fig. 1:**
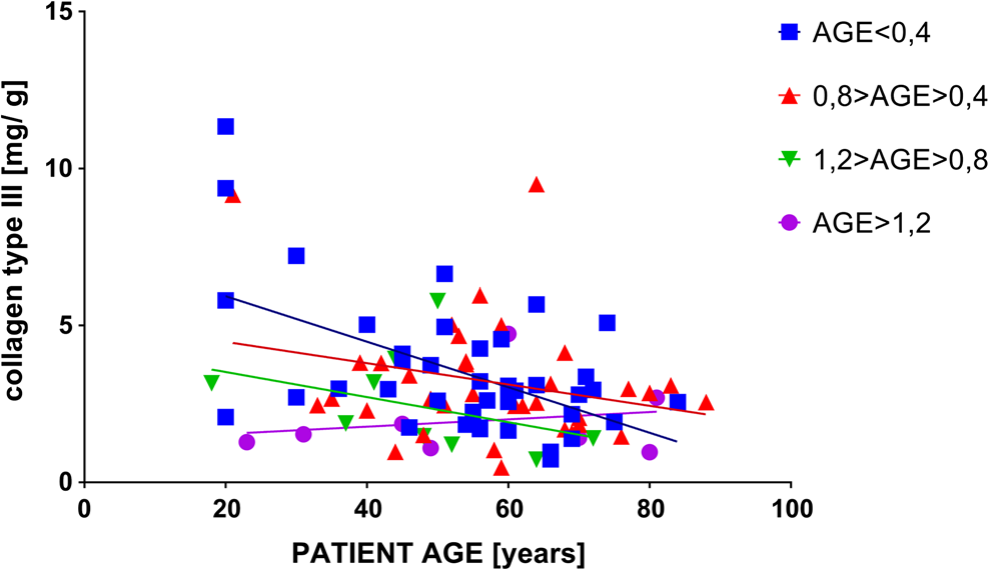
Graph showing the relationship between the three parameters: the age of the person from whom originates section of the aorta, the content of collagen type III and advanced glycation end products in the segment of the aorta

Additionally, a multivariate regression analysis was done to probe whether such parameters as collagen type III, AGE, and age of donors are linked independently. The results are listed in Table 3. Graphically, age of donors, collagen type III and AGE dependence we have shown on Fig. 1. As it is apparent from the graph, that at low AGE content there is a clear negative correlation between age and collagen type III, whereas at high AGE content in arteries, correlation changes the sign, but generally the correlation coefficient is close to 0.

**Table 3:**
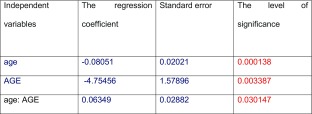
The results of multivariate regression analysis for the parameters: age of the person from whom originates section of the aorta, the content of collagen type III and AGE

**Fig. 2:**
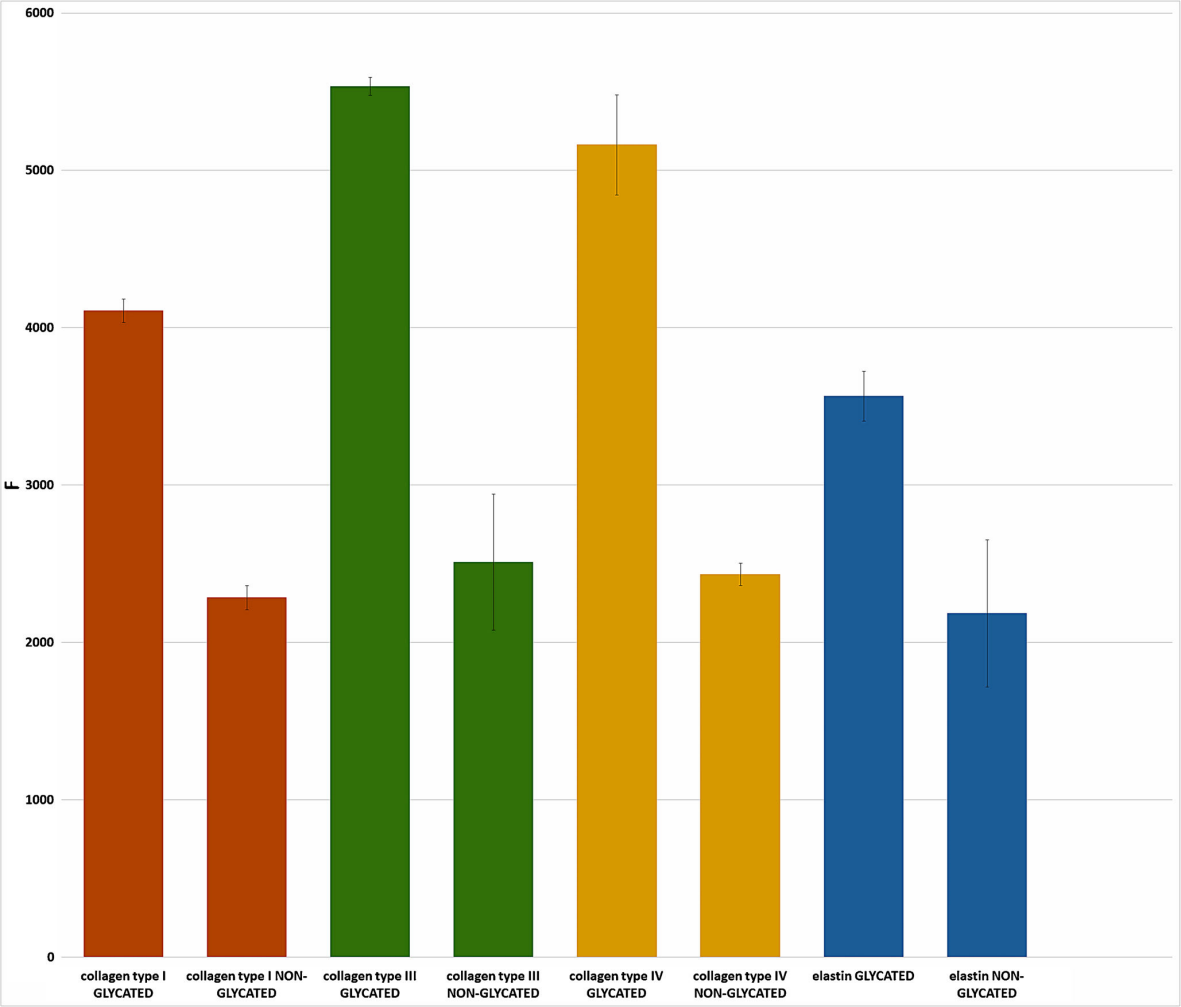
Fluorescence at wavelengths ex 350 and em 440 for collagen type I, III, IV and elastin samples subjected to glycation and untreated

**Fig. 3:**
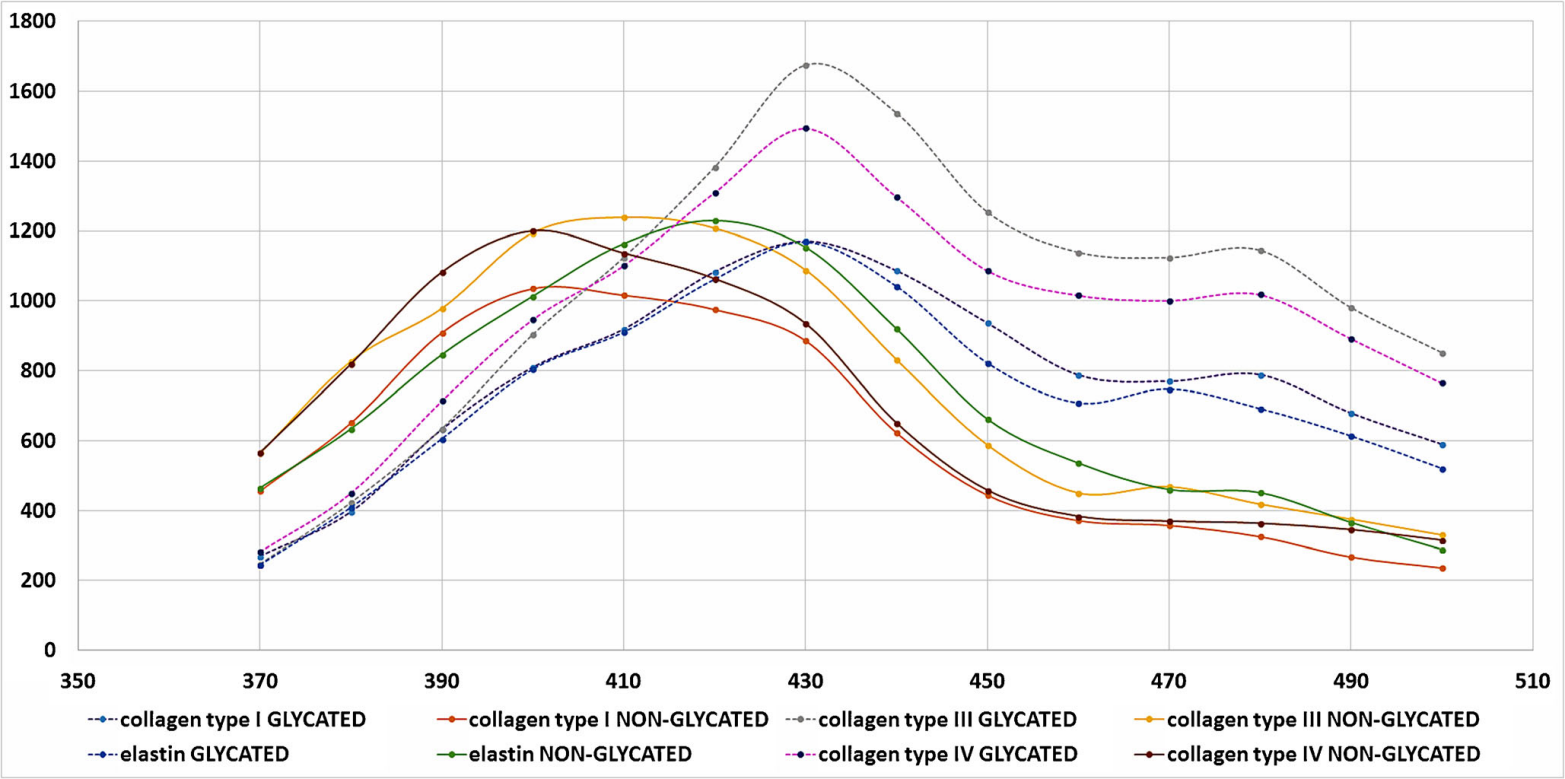
Fluorescence spectra for collagen types I, III, IV and elastin exposed to glycation and untreated at wavelength ex 350 and em 370–500

Glycated proteins fluoresce at wavelength of excitation 350 and emmision 440 approx. 50% more than proteins not subjected to glycation-induced conditions (Fig. 2). Also, the spectrum (ex 350 nm, em 370–500 nm) changes its course: glycated proteins have a spectrum that is clearly shifted towards longer wavelengths, with a clear peak at 430 nm, which is not observed for non-glycated proteins (Fig. 3).

**Fig. 4:**
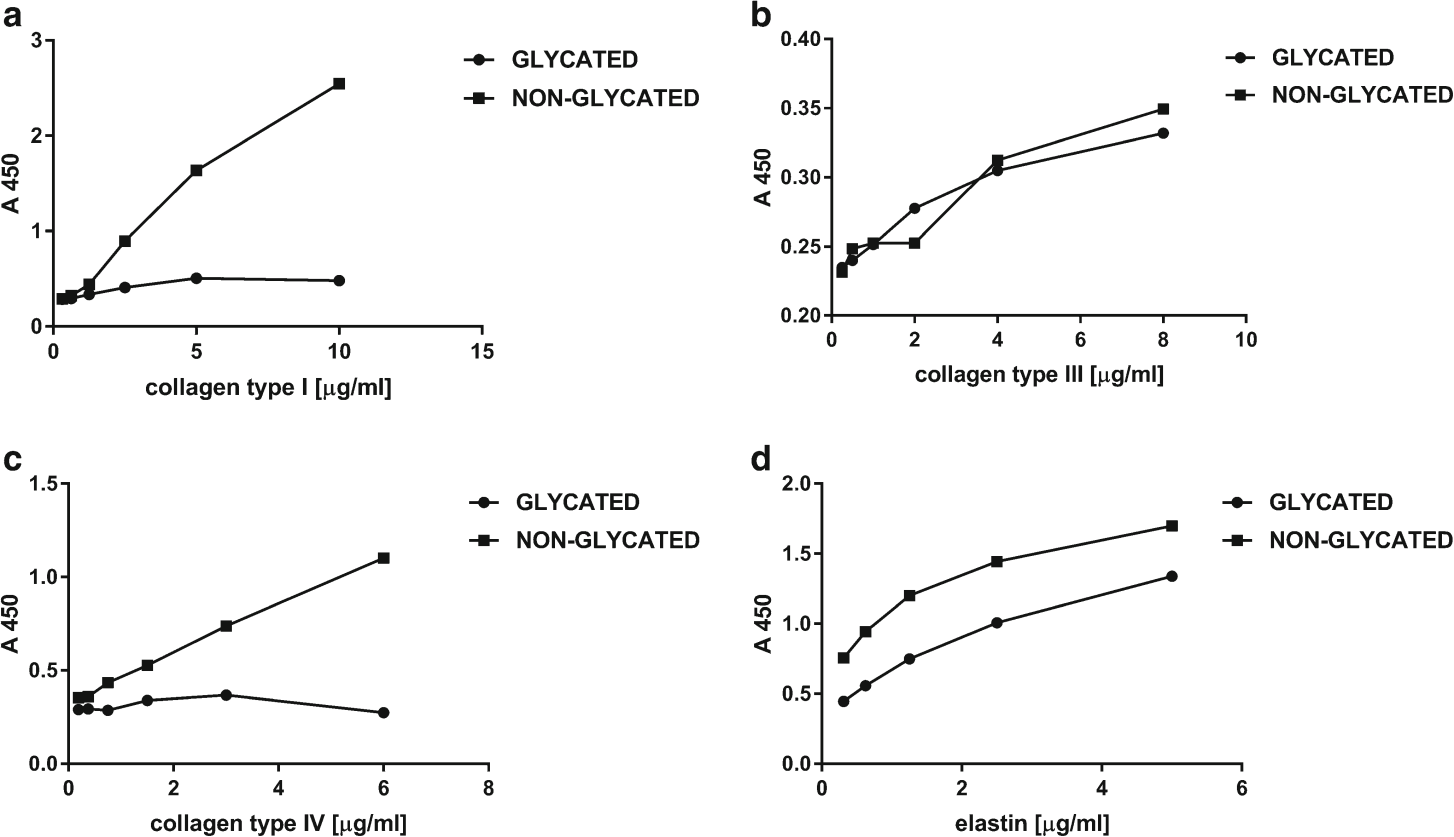
Standard curves of type I, III, IV collagen and elastin subjected and not subjected to glycation in the ELISA assay

Analysis of standard curves, where the test sample was subjected to conducive to glycation conditions compared to control showed significant differences between the glycated and controled assay, at least in the case of type I, IV collagen and elastin (Fig. 4). Much lower absorption was observed for the glycation probes, suggesting that the antibodies did not recognize the epitopes equally when the protein was glycated in comparison to non-glycated probes. The exception here is type III collagen, where both glycated and non-glycated proteins react in a similar way, resulting in the same, quite low, absorbances.

## Discussion

It is well established that the content of the extracellular matrix in the arteries grows with age, and increases stiffness of the blood vessels [6, 7].

It is also well known that with age amount of the atherosclerotic plaques increases and that proteolytic enzymes produced by macrophages and other cells, degrade ECM proteins, what intuitively should lead to a decrease in the content of collagen and elastin in the arteries [10].

The first assertion, concerning the increase in the content of collagen and elastin in the arteries, is based on the observation that with progress of the atherogenesis pool of the smooth muscle cells (SMC), which are expressing ECM proteins, is increasing [14]. It was demonstrated that degenerative changes occurring in arterial wall result in a transition of miocytes from the contractile to the synthesizing phenotype, what has consequences in the expression of more collagen type I and III, elastin, fibronectin, and proteins, that are normally not being expressed, including collagen type VIII, osteopontin and tenascin [15]. Korol and coworkers, while using a wide spectrum of methods (laser-induced fluorescence spectroscopy, gene expression profiling) demonstrated that in atherosclerotic plaques there is an increase in collagen type I and III [16]. Moreover, when using an animal model it was shown that lowering amount of fat in the diet reduced expression of MMP-1 and an increase in the amount of collagen in atherosclerotic plaques [17]. A similar phenomenon was observed when statins, drugs lowering blood lipids in hyperlipidemia, were applied. Statins reduced activity of collagenase and contributed to building a thicker layer of collagen in the arteries [18].

An alternative hypothesis is based on the observation that with age there is a reduction in the amount of collagen in arteries, and particularly during atherosclerosis. It is believed that degradation outweighs production of the extracellular matrix proteins during atherosclerosis. Responsible for this degradation are metalloproteinases, in particular MMP-1, gelatinases and stromelysins released from miocytes, macrophages, monocytes, lymphocytes, endothelial and other cells [19, 20]. It has been postulated that number of mast cells, T lymphocytes and macrophages containing MMP-9 increases with the severity of the plaque manifestation [21]. Furthermore, there are reports that thrombolytic therapy applied in the course of cardiovascular diseases causes the breakdown of collagen molecules [22, 23].

We have not observed a relationship between the content of collagen type I, IV, the sum of collagens in samples of aortas and the age of the donor. There was however a weak negative correlation between the content of collagen type III and age, suggesting that the amount of this protein decreases during aging. This result is consistent with findings by Tagasako and coworkers, who showed that a total amount of type III collagen in all tissues, including the arteries, decreases with age [24].

When considering a question of the relationship between the content of elastin in arteries and age of patients we should keep in mind that elastin is expressed mainly in the prenatal period and about natal, and its half-life is estimated at 40–70 years [4, 25]. Therefore, an obvious conclusion that comes to mind is that elastin’s amount should decrease by half after four decades of life. This argument is contradicted by the observation that with age quantity of elastin in the arteries is increasing. It appears that cells building vessel’s wall, such as miocytes, endothelial cells and fibroblasts, are capable of expressing ECM proteins in response to increased, age-related, mechanical stress [26].

While a number of studies provide evidence that the amount of elastin grows with age and progress of atherosclerosis [26–29]; nearly as many publications aim at proving a directly opposite hypothesis [5, 30–32]. There are also studies showing no link between amount of elastin in the arteries and age [33–36]. The latter literature findings agree with the results of this study, since no correlation between age and elastin content was found (Table 2).

In this work we have noted correlation between AGE and the content of type III and type IV collagens, both weak and negative. This is quite surprising, since one would expect that collagen, which is in fact a substrate for the glycation, should positively correlate with AGE, intuitively following the principle: the more of the substrate, the more product. Our results stand contrary to such an assumption, since with elevated content of the collagens type III and IV, less of AGEs were observed. After multiple regression analysis, and taking into account the parameter of age, it appears that with increasing age there is a decrease in correlation between AGE and collagen type III, or in another words: the low-AGE results show a negative correlation whereas, at high AGE in arteries correlation practically does not exist. A simple interpretation of these data leads to the conclusion that with increasing age the relationship between the amount of collagen type III and the age is in some way disturbed. The current state of knowledge does not allow to explain what factors influence this phenomenon.

Additional experience has been made on the glycated protein standards and the comparison with the curves made on non-glycated standards was done. It turned out that the curves are still linear or hyperbolic, but the absorbances for them are much smaller. Since the same antibodies were used at the same concentration, it can be concluded that these antibodies do not react with epitopes on glycated proteins in the same way as with unmodified proteins. This may in part be due to the denaturation of some of the molecules that have been stored for 11 days at 37 ° C, partly from glycation. Results for type III collagen for which both curves of glycated and unmodified protein overlap, where the absorbances are generally low, indicate that the protein was partially denatured or less prone to glycation compared to collagen type I, type IV of elastin.

Analysis of the above-mentioned results allows for some supposition that is a key to this work. We may have discovered the source of artifacts affecting determination of the relationship between the contents of collagen and elastin and age of the donor. Over time, the matrix proteins are steadilly being glycated. This leads to modification of the epitopes for anti-elastin and anti-collagen antibodies, decreasing the outcome of the immunological reaction. As mentioned in the introduction, glycation also results in the formation of the crosslinks and in a change of the electrostatic charges in proteins, which may influence the antigen-antibody interaction, or even block access of the antibody to the epitope.

The contradictory results obtained during analysis of the relationship between the content of ECM proteins and age, perhaps between the content of ECM proteins and severity of atherosclerosis (assuming that atherosclerosis can accelerate glycation) could have been caused by discounting the effect of glycation on the outcome of ELISA. This appears to be a novel observation, and to our best knowledge, has not yet been described in the literature. All studies that examine the collagen and elastin content with immuno-enzymatic methods (more often immunohistochemistry techniques, than ELISA) did not take into account protein glycation. When a diverse group of samples, both human and animal, representing different age groups is being tested, it is expected that degree of protein glycation in these samples will be different. A further complication may come a large group of donors with hyperglycemia related to diabetes. The intensity of protein glycation is affected by diabetes and also by other diseases that include atherosclerosis, Alzheimer’s disease, obesity, chronic inflammation and cancer [37, 38].

This is an important methodological problem, since the content of the individual ECM proteins will be underestimated to an unknown degree. Perhaps, to avoid such errors it would be beneficial to digest biological material by non-specific proteases, eg. proteinase K, to expose inaccessible epitopes, but this is likely to be insufficient because cross-bindings characteristic for glycation, as signaled, are not susceptible to enzymatic activity. It is important to continue research on this issue.

## Summary and conclusions

There is a lot of conflicting information in the literature describing collagen and elastin’s content in human arteries in relation to age. That chaos may originate from the fact that research groups use different techniques and experimental models (animal/human tissue, different sections of the arteries). It has not unequivocally been demonstrated whether these protein contents decrease or increase with age, although progressive stiffening of the arteries has been observed, suggesting that the amount of collagen increases and elastin decreases with age and progression of the degenerative processes in arteries. Nevertheless, our results show a decrease in the content of one of the fibrillar collagen types, type III, with age of the tissue donor.

We have found that analyzed specimens of arteries contained advanced glycation end-products at 0.522 (+/− 0.373) mg per 1 g of tissue. The AGE content negatively correlates with the amount of collagen type III and IV, although with increasing age the correlation between AGE and collagen type III decreases. Attention should be drawn to protein glycation as a potential source of artefacts that may affect the ECM proteins’ immunoenzymatic analysis. Analysis of samples, diverse in terms of the patient’s age and disease (*e.g*. diabetes), may involve significant error, which should be considered when interpreting the results.
